# Synthesis and Biological Evaluation of Novel Gigantol Derivatives as Potential Agents in Prevention of Diabetic Cataract

**DOI:** 10.1371/journal.pone.0141092

**Published:** 2015-10-30

**Authors:** Jie Wu, Chuanjun Lu, Xue Li, Hua Fang, Wencheng Wan, Qiaohong Yang, Xiaosheng Sun, Meiling Wang, Xiaohong Hu, C.-Y. Oliver Chen, Xiaoyong Wei

**Affiliations:** 1 School of Basic Medical Sciences, Guangzhou University of Chinese Medicine, Guangzhou, 510006, China; 2 College of Chemical Engineering, Zhejiang University of Technology, Hangzhou, 310014, China; 3 Institute of Drug Synthesis and Pharmaceutical Processing, School of Pharmaceutical Sciences, Sun Yat-sen University, Guangzhou, 510006, China; 4 Antioxidants Research Laboratory, Jean Mayer USDA Human Nutrition Research Center on Aging, Tufts University, Boston, MA, 02111, United States of America; Univeristy of Miami, UNITED STATES

## Abstract

As a continuation of our efforts directed towards the development of natural anti-diabetic cataract agents, gigantol was isolated from Herba dendrobii and was found to inhibit both aldose reductase (AR) and inducible nitric oxide synthase (iNOS) activity, which play a significant role in the development and progression of diabetic cataracts. To improve its bioefficacy and facilitate use as a therapeutic agent, gigantol (compound **14f**) and a series of novel analogs were designed and synthesized. Analogs were formulated to have different substituents on the phenyl ring (compounds **4**, **5**, **8**, **14a-e**), substitute the phenyl ring with a larger steric hindrance ring (compounds **10**, **17c**) or modify the carbon chain (compounds **17a**, **17b**, **21**, **23**, **25**). All of the analogs were tested for their effect on AR and iNOS activities and on D-galactose-induced apoptosis in cultured human lens epithelial cells. Compounds **5**, **10**, **14a**, **14b**, **14d**, **14e**, **14f**, **17b**, **17c**, **23**, and **25** inhibited AR activity, with IC_50_ values ranging from 5.02 to 288.8 μM. Compounds **5**, **10**, **14b**, and **14f** inhibited iNOS activity with IC_50_ ranging from 432.6 to 1188.7 μM. Compounds **5**, **8**, **10**, **14b**, **14f**, and **17c** protected the cells from D-galactose induced apoptosis with viability ranging from 55.2 to 76.26%. Of gigantol and its analogs, compound **10** showed the greatest bioefficacy and is warranted to be developed as a therapeutic agent for diabetic cataracts.

## Introduction

Gigantol (4-[2-(3-hydroxy-5-methoxyphenyl)ethyl]-2-methoxyphenol, PubChem CID: 10221179) is a naturally occurring 1,2-diphenylethane(bibenzyl) found in Herba dendrobii [1]. The literature has shown that gigantol has several bioactions, e.g. anti-carcinogenic [2–5], antioxidant [6], anti-aging [7], anti-coagulating [8], anti-mutagenic [9], antispasmodic [10–12], and anti-inflammatory [13]. Although the structure of gigantol is different from that of more extensively studied aldose reductase (AR) inhibitors, such as carboxylic acids, spirohydantoin derivatives, and compounds with sulfonyl groups [14–16]. Previous studies have shown that gigantol extracted from dendrobii prevented and inhibited development of cataracts through its inhibitory effect on the activity of AR and inducible nitric oxide synthase (iNOS) [17].

Cataracts are the leading cause of visual impairment and blindness worldwide [18]. The development and progression of cataracts are attributed to a wide range of risk factors, e.g. aging, genetics, radiation, medications, and diseases. Among these factors, chronic hyperglycemia is understood to increase the risk of cataracts because hyperglycemic conditions increase osmotic pressure and induce oxidative damage in lenses, partially through the activation of AR and iNOS [19–22]. AR converts glucose to sorbitol, whose accumulation inside cells in turn causes fluid accumulation, elevates osmotic pressure, and induces lens swelling and degeneration of hydropic lens fibers [23–25]. All of these events enable cataract development. Furthermore, peroxynitrites are formed from superoxides and nitric oxides when iNOS expression and activity is up-regulated by the hyperglycemic condition involved in pathogenesis of cataracts [26].

Due to increasing number of patients with diabetes worldwide, the incidence of diabetic cataracts is steadily increasing [27]. Even though cataract surgery is an effective cure, this operation may not be the best option for all patients because of surgery related health concerns, complications, and costs [28, 29]. For this reason, it is necessary to develop pharmacological therapies for diabetic cataract treatment and prevention. In this context, gigantol could be a suitable drug candidate for the treatment and prevention of diabetic cataracts. However, the limited availability of gigantol from its natural source, Herba dendrobii and other plants, may limit its development and use in diabetic cataract prevention. Thus, to continue investigating applicability of gigantol in diabetic cataracts, chemical synthesis of gigantol and its analogs becomes a viable approach. In addition to serving as a therapeutic agent for diabetic cataracts, some of these analogs could be valuable drug candidates for tumor therapy, local anesthetics, antidepressants, or antipsychotics, and smooth muscle relaxants [30]. Because the bioactivity and bioefficacy of these analogs have not been assessed in diabetic cataracts, the main objective of the study was to synthesize gigantol and its analogs and then assess their effect on the development and progression of diabetic cataracts through modulation of AR and iNOS. The gigantol analogs were synthesized by using different substituents on the phenyl ring (compounds **4**, **5**, **8**, **14a–e**), substituting the phenyl ring with a larger steric hindrance ring (compounds **10**, **17c**), and changing the carbon chain (compounds **17a**, **17b**, **21**, **23**, **25**). Their bioactions were assessed by determining their capability to inhibit AR and iNOS activity and ameliorate D-galactose-induced death of cultured human lens epithelial cells (HLECs).

## Results and Discussion

### Synthesis of gigantol and its analogs

**Fig 1:**
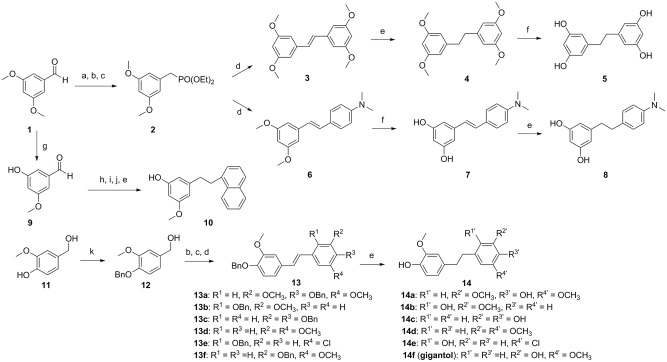
Synthesis of 4, 5, 8, 10, 14, and gigantol. Reagents and conditions: a. NaBH_4_, MeOH; b. PBr_3_, pyridine, 0°C; c. P(OEt)_3_, 120°C; d. different aldehydes, CH_3_ONa, 0°C to room temperature (RT), 12 h; e. Pd/C, H_2_, RT, 12 h; f. BBr_3_, CH_2_Cl_2_, -20°C, 2 h; RT, 4 h; g. NaH, ethanethiol, DMF, N_2_, reflux; h. MOMCl, *i*-Pr_2_NEt, CH_2_Cl_2_, 0°C, 1 h; RT, 12 h; i. diethyl naphthalen-1-ylmethylphosphonate, CH_3_ONa, 0°C, 1 h; rt, 12 h; j. 2 M HCl, methanol, 50°C, 1 h; k. BnBr, 18-crown-6, K_2_CO_3_, reflux, 9 h.

**Fig 2:**
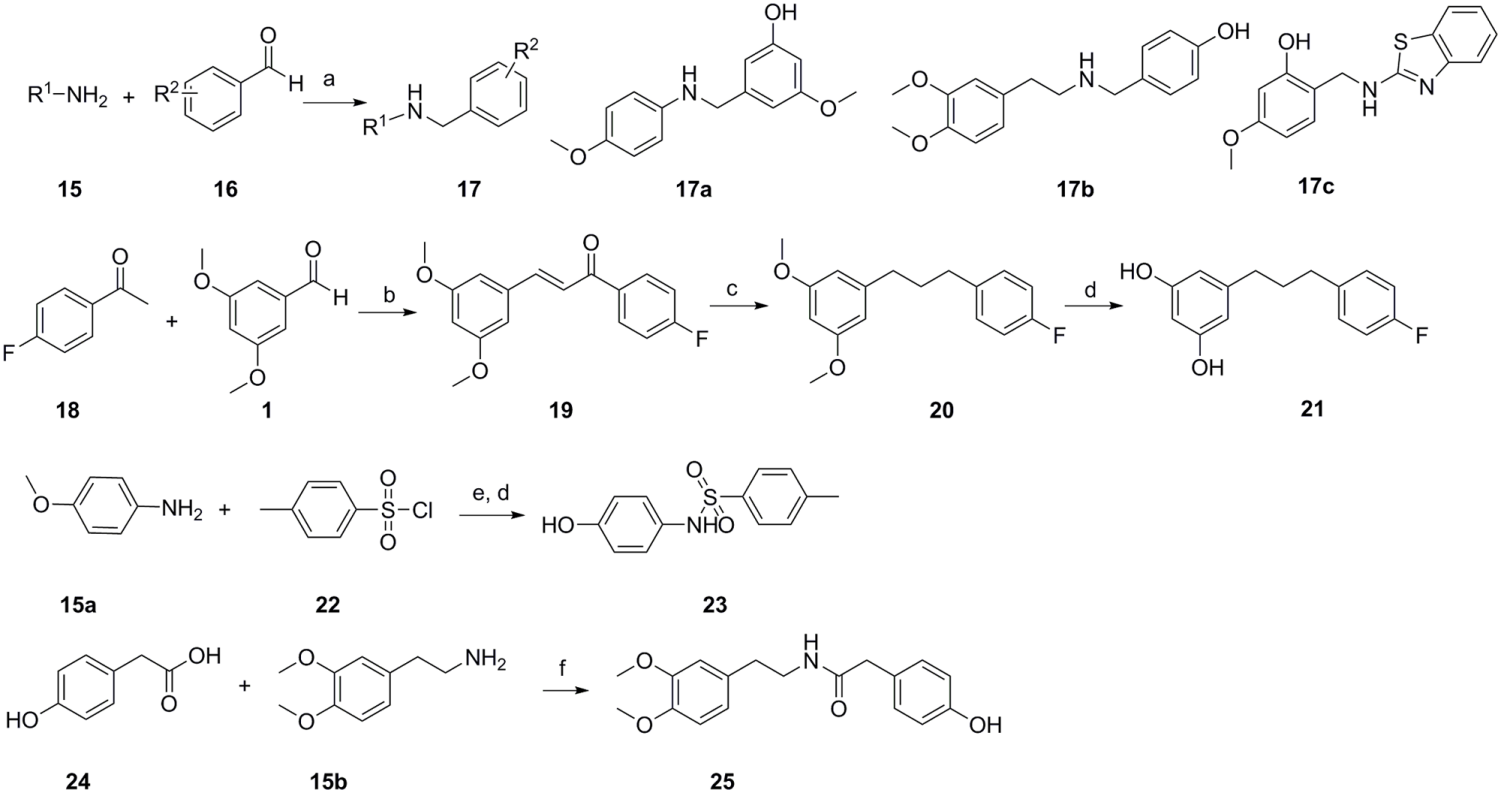
Synthesis of 17, 21, 23, and 25. Reagents and conditions: a. TsOH, ethanol; 0°C, NaBH_4_; b. K_2_CO_3_, ethanol; c. Pd/C, H_2_, RT, 12 h; d. BBr_3_, CH_2_Cl_2_, -20°C, 2 h; RT, 4 h. e. Et_3_N, CH_2_Cl_2_; f. 180°C, neat, N_2_.

The routes of synthesis of gigantol analogs are shown in Figs 1 and 2. Compounds **5** and **8** were synthesized in six steps according to previously reported procedures (Fig 1) [31]. Using commercially available 3,5-dimethoxybenzaldehyde as the starting material, compound **2** was synthesised through reduction, bromination, and reaction with triethylphosphite. Compound **2** served as the starting compound. Wittig olefination, followed by hydrogenation and demethylation, produced compounds **5** and **8**. The synthesis of compounds **10**, **14**, and **14f** was similar to that of compound **4**, except that the starting material was first protected by chloromethyl methyl ether (MOMCl) and benzyl bromide, respectively (Fig 1). Compounds **17a–c** were synthesized in one pot. Amine reacted with aldehyde to produce imine, and NaBH_4_ was then added to produce the target compounds (Fig 2). Intermediate compound **19** was synthesized by aldol condensation, followed by hydrogenation and demethylation to yield compound **21** (Fig 2). As shown in Fig 2, compound **23** was generated by reacting 4-methoxyaniline with 4-methylbenzene-1-sulfonyl chloride followed by the addition of BBr_3_. Compound **25** was produced by the reaction of 2-(4-hydroxyphenyl)acetic acid and 2-(3,4-dimethoxyphenyl)ethanamine with stirring at 180°C without solvent under N_2_. The purity of all synthesized compounds was determined by HPLC.

### Biological activities of gigantol and analogs

#### Evaluation of AR inhibitory properties

AR has been acknowledged as a validated diabetic cataract inducer [32–36]. Thus, we tested the potential of gigantol and its analogs for prevention and treatment of diabetic cataract by assessing their capability to inhibit AR activity. Table 1 shows that compounds **14a–e** were more potent than synthetic gigantol (compound **14f**) except compound **14c**. Results showed that most synthetized compounds were capable of inhibiting AR activity with their IC_50_ values ranging from 5.02 to 347.35 μM in a dose-dependent manner, which were at least 5 times lower than the extracted gigantol. Among these synthetic compounds, compounds **23** (5.02 μM), **14a** (17.28 μM), **14e** (21.83 μM), and **10** (31.86 μM) displayed potency in AR inhibition [37, 38]. Of all tested compounds, sulfonamide compound **23** appeared to be the best inhibitor, suggesting that the *N*-sulfonylation link might play a critical role in the binding of the compound to the AR catalytic site because sulfonyl group has been reported as an important pharmacophore of AR inhibitors [39–42]. As synthetic gigantol (compound **14f**) exhibited intermediate potency, we found that substituting one of the phenyl ring in gigantol with a larger steric hindrance naphthalene ring made the compound **10** 9-fold more potent. In order to study the role of the 4-hydroxy-3-methoxyphenyl ring in the AR inhibition, we synthesized compounds **14a-e** by keeping 4-hydroxy-3-methoxyphenyl ring and placing different substituents on the other phenyl ring, and results showed that significance of the 4-hydroxy-3-methoxyphenyl ring in AR inhibition. These results suggest that compounds **10**, **14a**, **14e**, and **23** can be considered as lead compounds for further development of new diabetic cataract drugs.

**Table 1:** Inhibitory effect of gigantol and its analogs on AR activity^1^.

Compound	IC_50_ (μM)	Compound	IC_50_ (μM)
Extractive gigantol	2516.6 ± 10.35	**14e**	21.83 ± 5.47
**4**	347.35 ± 3.74	**14f**	176.06 ± 3.21*
**5**	288.80 ± 2.16	**17a**	NA
**8**	513.38 ± 2.33	**17b**	173.73 ± 3.38
**10**	31.17 ± 1.51	**17c**	242.67 ± 5.67
**14a**	17.28 ± 1.72	**21**	556.34 ± 4.37
**14b**	125.94 ± 1.3	**23**	5.02 ± 2.57
**14c**	534.35 ± 5.44	**25**	54.44 ± 2.39
**14d**	39.20 ± 2.13		

^1^The results are expressed as mean ± SD (n = 3).

Abbreviation: NA, no activity

*P < 0.01, vs. Extractive gigantol.

#### Assessment of anti-iNOS inhibitory properties

The role of iNOS in the development of diabetic cataracts has been well documented [21]. Results showed that compounds **5**, **10**, **14b**, and **14f** inhibited iNOS in a dose-dependent manner with IC_50_ values ranging from 432.6 to 1188.7 μM (Table 2). Although the IC_50_ of compounds **5**, **10**, **14b**, and **14f** was larger than that of the extracted gigantol, these compounds remain good candidates for the development of diabetic cataract drugs because of their superior AR inhibitory effect.

**Table 2:** Inhibitory effect of gigantol and its analogs on iNOS activity^1^.

Compound	IC_50_ (μM)	Compound	I C_50_ (μM)
Extractive gigantol	32.23 ± 2.61	**14e**	NA
**4**	NA	**14f**	680.07 ± 3.28
**5**	432.6 ± 2.37	**17a**	NA
**8**	NA	**17b**	NA
**10**	1188.7 ± 3.31	**17c**	NA
**14a**	NA	**21**	NA
**14b**	433.57 ± 4.23	**23**	NA
**14c**	NA	**25**	NA
**14d**	NA		

^1^The results are expressed as mean ± SD (n = 3). Abbreviation: NA, no activity.

### Evaluation of the effects on D-galactose-induced cell death in HLECs

**Fig 3:**
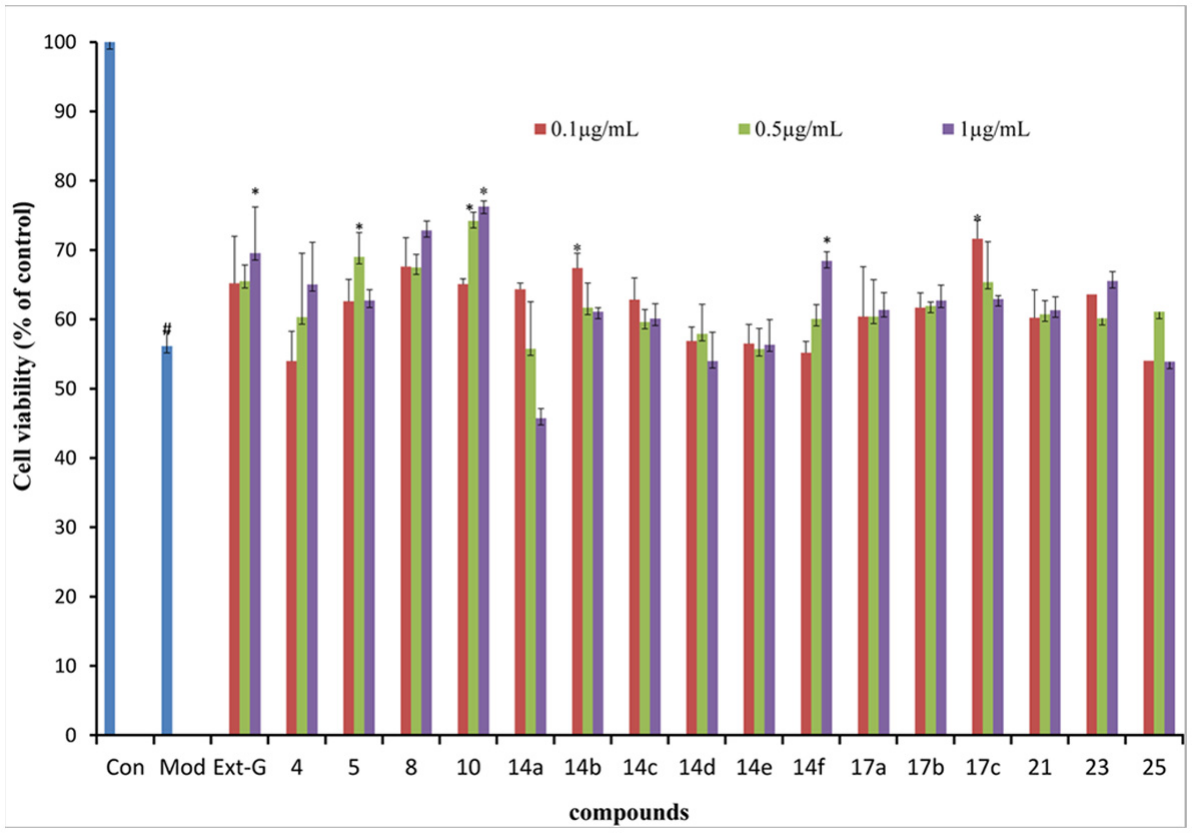
Gigantol analogs at 0.1, 0.5, and 1.0 μg·mL^-1^ on viability of HLECs treated with 250 mmol·L^-1^ D-galactose for 72 h. Cell viability was determined by the MTT assay in the absence (Con) and presence (all other groups) of D-galactose. Ext-G refers to gigantol extracted from dendrobii. Viability (mean ± SD, n = 3) is expressed as the percentage of viable cells in the treatment to those of the Con. ^#^ *P* < 0.01 vs. Con, *P < 0.05 vs. D-galactose.

Galactose toxicity causes the sequential death of different LEC populations in the lenses of galactosemic rats, starting with those in the central and peripheral mitotic zone, followed by the central (non-mitotic) LECs, and eventually the remaining LECs [43, 44]. In this study, the protective effect of the synthetic compounds was tested at concentrations 0.1, 0.5, and 1 μg·mL^-1^ on D-galactose-induced apoptosis of the cultured HLECs. Results showed that extracted gigantol (1.0 μg·mL^-1^, 5.28 μM)) and compounds **5** (0.5 μg·mL^-1^, 1 μM), **8** (1.0 μg·mL^-1^, 4.39 μM), **10** (0.5 μg·mL^-1^, 0.89 μM), **14b** (0.1 μg·mL^-1^, 0.366 μM), **14f** (1.0 μg·mL^-1^, 5.28 μM), and **17c** (0.1 μg·mL^-1^, 0.388 μM) protected HLECs from apoptosis. Of all compounds tested, compound **10** showed the most efficacious protection against apoptosis with the cell survival reaching 72.26% (*P*<0.05) (Fig 3). Given that apoptosis of HLEC contributes greatly to cataract formation, protecting HLECs against programmed cell death appears to be one of the therapeutic strategies for cataract treatment [45]. Results show that extracted gigantol and compounds **5**, **8**, **10**, **14b**, **14f**, and **17c** were the most effective in the protection of LEC against D-galactose-induced apoptosis.

## Conclusions

Gigantol and its synthetic analogs were evaluated for their potential to treat diabetic cataracts. Synthetic compounds were designed by placing different substituents on the phenyl ring (compounds **4**, **5**, **8**, **14a-e**), substituting the phenyl ring with a larger steric hindrance ring (compounds **10**, **17c**), and changing the carbon chain (compounds **17a**, **17b**, **21**, **23**, **25**). We found compounds **4**, **5**, **10**, **14b**, **14d**, **14e**, **14f**, **17c**, **23**, and **25** inhibited AR activity with the IC_50_ value ranging from 5.02 to 347.35 μM and compounds **5**, **10**, **14b**, and **14f** inhibited iNOS activity with the IC_50_ values ranging from 432.6 to 1188.7 μM. Furthermore, compounds **5**, **8**, **10**, **14b**, **14f**, and **17c** and the extracted gigantol protected HLECs from apoptosis. Among all of the test compounds, only synthesized gigantol and its analogs **5**, **10**, and **14b** were effective to inhibited AR and iNOS activities and reduced HLEC apoptosis. Of these 3 compounds, compound **10** (3-methoxy-5-(2-(naphthalen-1-yl)ethyl)phenol) showed the most pronounced protective effect against apoptosis, and further studies are needed to test its candidacy as a diabetic cataract drug, starting with the bioavailability, efficacy, and toxicity in *in vivo* experiments.

## Materials and Methods

### Synthesis

Mass spectrometry was performed on an Agilent LC–MS 6120 system equipped with an ESI mass spectrometer. Melting points were determined using an SRS OptiMelt Automated Melting Point System. NMR spectra were generated on a Bruker Avance III spectrometer (S1 File), using tetramethylsilane(TMS) as an internal standard. The purity of synthesized compounds was verified by an HPLC system equipped with an Eclipse Plus C8 column (4.6 × 150 mm, 5 μm). Compound **12** was synthesized according to the previously reported procedure [46].

#### General procedure 1

Phosphate (1.5 equiv.) and sodium methoxide (3 equiv.) were mixed in DMF and stirred for 30 min at 0°C. Then, 1 equiv. aldehyde was added to DMF under nitrogen. The resulting mixture was stirred overnight at room temperature, quenched by addition of ice-cold water, and extracted using ethyl acetate. After the removal of organic solvents, the crude product was purified using silica gel chromatography with ethyl acetate/petroleum ether as the eluent.

#### General procedure 2

To compound dissolved in methanol, 10% Pd/C was added and the resulting mixture was stirred overnight at room temperature under hydrogen. The weight of compound to Pd/C was 10:1. After the reaction mixture was filtrated and concentrated, the crude product was purified either by recrystallization or silica gel chromatography to yield the target product.

#### General procedure 3

BBr_3_ (4 equiv.) was added dropwise to a 1 equiv. solution of compound in dried CH_2_Cl_2_ at -20°C under nitrogen. The resulting solution was slowly warmed to room temperature and stirred overnight. Then, ice-cold water was slowly added and the mixture filtered to yield the crude product. The final product was obtained after purification using recrystallization or silica gel chromatography.

#### General procedure 4

*p*-Toluenesulfonic acid (0.2 equiv) was added to a solution of amine (1.0 equiv) and aldehyde (1.0 equiv) in ethanol, and the resulting mixture was stirred at room temperature. When the raw material faded (as monitored by TLC), NaBH_4_ (3.0 equiv) was added at 0°C. The mixture was stirred for 1 h, followed by addition of water and extraction using ethyl acetate. Finally, all solvents were removed under reduced pressure to obtain the crude product, which was purified by flash chromatography on silica gel.

#### (*E*)-1,2-bis(3,5-dimethoxyphenyl)ethane (3)

Compound **3** was synthesized according to the general procedure 1. The crude product was purified by column chromatography (petroleum ether: ethyl acetate = 30:1) to yield **3** (0.51 g, 85%) as a white solid. ^1^H NMR (400 MHz, CDCl_3_) δ 7.01 (s, 2H), 6.67 (d, *J* = 2.2 Hz, 4H), 6.41 (t, *J* = 2.2 Hz, 2H), 3.83 (s, 12H). ^13^C NMR (101 MHz, CDCl_3_) δ 161.0, 139.2, 129.2, 104.7, 100.2, 55.4. LC/MS (ESI): 254.1[M+H]^+^. HPLC purity: 97.1%.

#### 1,2-bis(3,5-dimethoxyphenyl)ethane (4)

Compound **4** was synthesized according to the general procedure 2. The crude product was purified by column chromatography (petroleum ether: ethyl acetate = 40:1) to yield **4** (0.26 g, 87%) as a white solid. mp: 104.6–107.9°C. ^1^H NMR (400 MHz, CDCl_3_) δ 6.36–6.35 (m, 4H), 6.33–6.31 (m, 2H), 3.77 (d, *J* = 1.1 Hz, 12H), 2.85 (s, 4H). ^13^C NMR (101 MHz, CDCl_3_) δ 160.8, 144.1, 106.5, 98.0, 55.3, and 38.0. LC/MS (ESI): 303.1 [M+H]^+^. HPLC purity: 96.3%.

#### 5,5'-(ethane-1, 2-diyl)dibenzene-1,3-diol5,5'-(ethane-1,2-diyl)dibenzene -1,3-diol (5)

Compound **5** was synthesized according to the general procedure 3. The crude product was purified by recrystallization with MeOH to yield **5** (0.11 g, 68%) as a white solid. mp: 245.9–248.1°C. ^1^H NMR (400 MHz, MeOD): δ 6.15 (s, 4H), 6.09 (s, 2H), 2.69(s, 4H). ^13^C NMR (101 MHz, MeOD): δ 159.3, 145.6, 108.0, 101.2, 39.0. LC/MS (ESI): 247.1 [M+H]^+^. HPLC purity: 99.4%.

#### 5-4-(dimethylamino)phenethyl)benzene-1,3-diol (8)

Compound **8** was synthesized according to previously reported procedures [32]. The crude product was purified over silica gel column chromatography (petroleum ether: ethyl acetate = 5:1) yielding **8** (0.09 g, 90%) as a gray solid. mp: 152.5–154.1°C. ^1^H NMR (400 MHz, MeOD) δ 7.29 (d, *J* = 8.6 Hz, 2H), 7.22 (d, *J* = 8.6 Hz, 2H), 5.99 (dd, *J* = 5.7, 1.6 Hz, 3H), 3.10 (s, 6H), 2.85–2.76 (m, 2H), 2.65 (t, *J* = 7.6 Hz, 2H). ^13^C NMR (101 MHz, MeOD) δ 159.4, 144.9, 143.7, 143.3, 131.5, 120.3, 108.2, 101.4, 46.4, 38.6, 37.8. LC/MS (ESI): 258.1 [M+H]^+^. HPLC purity: 97.4%.

#### 3-hydroxy-5-methoxybenzaldehyde (9)

Compound **9** was synthesized using previously reported procedures [47]. The characteristic of the compound is white solid and mp: 67–68°C, the yield was 53%. ^1^H NMR (400 MHz, MeOD) δ 9.83 (s, 1H), 6.93 (ddd, *J* = 14.6, 2.2, 1.3 Hz, 2H), 6.65 (t, *J* = 2.3 Hz, 1H), 3.82 (s, 3H). ^13^C NMR (101 MHz, MeOD) δ 194.0, 162.9, 160.6, 140.1, 110.0, 108.8, 106.7, 56.0.

#### 3-methoxy-5-(2-(naphthalen-1-yl)ethyl)phenol (10)

Compound **10** was synthesized with compound **9** according to the general procedure 1 after protecting by MOMCl, following by deprotection and hydrogenation to produce the title product. The crude product was purified over silica gel column chromatography (petroleum ether: ethyl acetate = 2:1) yielding **10** (0.20 g, 72%) as a white solid. mp: 103.9–106.7°C. ^1^H NMR (400 MHz, CDCl_3_) δ 8.08 (d, *J* = 8.1 Hz, 1H), 7.87 (d, *J* = 7.6 Hz, 1H), 7.73 (d, *J* = 8.1 Hz, 1H), 7.60–7.44 (m, 2H), 7.39 (t, *J* = 7.6 Hz, 1H), 7.30 (d, *J* = 6.9 Hz, 1H), 6.45–6.21 (m, 3H), 4.87 (br, 1H), 3.76 (s, 3H), 3.46–3.24 (m, 2H), 3.08–2.85 (m, 2H). ^13^C NMR (101 MHz, CDCl_3_) δ 160.9, 156.8, 144.8, 137.7, 133.9, 131.8, 128.9, 126.8, 126.0, 125.9, 125.6, 125.5, 123.6, 108.0, 106.8, 99.2, 55.3, 37.1, 34.7. LC/MS (ESI): 279.1 [M+H]^+^. HRMS calcd for C_19_H_18_O_2_ [M+H]^+^: 279.1380, found: 279.1381. HPLC purity: 95.0%.

#### (*E*)-2-(benzyloxy)-5-(4-(benzyloxy)-3-methoxystyryl)-1,3-dimethoxybenzen (13a)

Compound **13a** was synthesized according to the general procedure 1. The crude product was purified by column chromatography (petroleum ether: ethyl acetate = 20:1) to yield **13a** (0.25 g, 52%) as a white solid. ^1^H NMR (400 MHz, CDCl_3_) δ 7.49 (d, *J* = 6.9 Hz, 2H), 7.45 (d, *J* = 7.2 Hz, 2H), 7.34 (ddd, *J* = 22.4, 13.2, 7.1 Hz, 6H), 7.07 (d, *J* = 1.7 Hz, 1H), 7.01–6.95 (m, 1H), 6.89 (dd, *J* = 18.3, 8.4 Hz, 3H), 6.70 (s, 2H), 5.18 (s, 2H), 5.02 (s, 2H), 3.95 (s, 3H), 3.87 (s, 6H).

#### (*E*)-2-(benzyloxy)-1-(4-(benzyloxy)-3-methoxystyryl)-3-methoxybenzene (13b)

Compound **13b** was synthes-ized according to the general procedure 1. The crude product was purified by column chromatography (petroleum ether: ethyl acetate = 15:1) to yield **13b** (0.15 g, 33%) as a white solid. ^1^H NMR (400 MHz, CDCl_3_) δ 7.53–7.46 (m, 2H), 7.46–7.41 (m, 2H), 7.41–7.26 (m, 7H), 7.21 (d, *J* = 7.9 Hz, 1H), 7.06 (q, *J* = 8.0 Hz, 1H), 7.02–6.95 (m, 2H), 6.86 (ddd, *J* = 8.8, 8.2, 6.8 Hz, 3H), 5.16 (s, 2H), 5.01 (s, 2H), 3.89 (s, 3H), 3.87 (s, 3H).

#### (*E*)-(4-(4-(benzyloxy)-3-methoxystyryl)-1,2-phenylene)bis(oxy)bis(methylene) dibenzene (13c)

Compound **13c** was synthesized according to the general procedure 1. The crude product was purified by column chromatography (petroleum ether: ethyl acetate = 10:1) to yield **13c** (0.24 g, 45%) as a white solid. ^1^H NMR (400 MHz, CDCl_3_) δ 7.50–7.41 (m, 6H), 7.41–7.28 (m, 9H), 7.12 (s, 1H), 7.04 (s, 2H), 6.92 (s, 2H), 6.85 (s, 3H), 5.20 (s, 2H), 5.17 (s, 4H), 3.94 (s, 3H).

#### (*E*)-1-(benzyloxy)-4-(3,5-dimethoxystyryl)-2-methoxybenzene (13d)

Compound **13d** was synthesized according to general procedure 1. The crude product was purified by column chromatography (petroleum ether: ethyl acetate = 10:1) to yield **13d** (0.15 g, 40%) as a white solid. ^1^H NMR (400 MHz, CDCl_3_) δ 7.44 (d, *J* = 7.1 Hz, 2H), 7.37 (dd, *J* = 8.1, 6.6 Hz, 2H), 7.31 (d, *J* = 7.2 Hz, 1H), 7.08 (d, *J* = 1.9 Hz, 1H), 6.99 (dd, *J* = 17.3, 9.1 Hz, 2H), 6.92–6.85 (m, 2H), 6.65 (d, J = 2.2 Hz, 2H), 6.38 (t, *J* = 2.2 Hz, 1H), 5.18 (s, 2H), 3.95 (s, 3H), 3.83 (s, 6H).

#### (*E*)-1-(benzyloxy)-2-(4-(benzyloxy)-3-methoxystyryl)-4-chlorobenzene (13e)

Compound **13e** was synthesized according to the general procedure 1. The crude product was purified by column chromatography (petroleum ether: ethyl acetate = 10:1) to yield **13e** (0.21 g, 46%) as a white solid. ^1^H NMR (400 MHz, CDCl_3_) δ 7.54 (d, *J* = 2.5 Hz, 1H), 7.44 (d, *J* = 7.4 Hz, 4H), 7.41–7.26 (m, 7H), 7.09 (ddd, *J* = 16.4, 7.9, 4.0 Hz, 3H), 7.01–6.93 (m, 1H), 6.85 (d, J = 8.5 Hz, 2H), 5.17 (s, 2H), 5.12 (s, 2H), 3.91 (s, 3H).

#### 4-(4-hydroxy-3-methoxyphenethyl)-2,6-dimethoxyphenol (14a)

Compound **14a** was synthesized according to the general procedure 2. The crude product was purified by column chromatography (petroleum ether: ethyl acetate = 5:1) to yield **14a** (0.10 g, 66%) as a blue solid. mp: 78.2–79.5°C. ^1^H NMR (400 MHz, CDCl_3_) δ 6.83 (d, *J* = 7.9 Hz, 1H), 6.67 (d, *J* = 7.9 Hz, 1H), 6.61 (s, 1H), 6.35 (s, 2H), 5.52 (br, 2H), 3.82 (d, *J* = 3.6 Hz, 9H), 2.81 (s, 4H). ^13^C NMR (101 MHz, CDCl_3_) δ 146.9, 146.3, 143.8, 133.7, 132.92, 132.88, 121.1, 114.3, 111.4, 105.3, 56.3, 55.9, 38.4, 37.9. LC/MS (ESI): 305.1 [M+H]^+^. HPLC purity: 99.7%.

#### 4-(2-hydroxy-3-methoxyphenethyl)-2-methoxyphenol (14b)

Compound **14b** was synthesized according to the general procedure 2. The crude product was purified by column chromatography (petroleum ether: ethyl acetate = 5:1) to yield **14b** (0.09 g, 99%) as a brown oil. ^1^H NMR (400 MHz, CDCl_3_) δ 6.82 (d, *J* = 8.0 Hz, 1H), 6.72 (ddd, *J* = 8.9, 8.3, 3.9 Hz, 5H), 5.77 (br, 1H), 5.53 (br, 1H), 3.86 (s, 3H), 3.82 (s, 3H), 2.96–2.79 (m, 4H). ^13^C NMR (101 MHz, CDCl_3_) δ 146.4, 146.3, 143.7, 143.6, 134.4, 127.8, 122.5, 121.1, 119.3, 114.2, 111.3, 108.6, 56.0, 55.9, 35.7, 32.3. LC/MS (ESI): 273.0 [M-H]^-^. HPLC purity: 95.0%.

#### 4-(4-hydroxy-3-methoxyphenethyl)benzene-1,2-diol (14c)

Compound **14c** was synthesized according to the general procedure 2. The crude product was purified by column chromatography (petroleum ether: acetone = 5:1) to yield **14c** (0.06 g, 53%) as a red oil. ^1^H NMR (400 MHz, CDCl_3_) δ 6.82 (d, *J* = 8.0 Hz, 1H), 6.76 (d, *J* = 8.0 Hz, 1H), 6.66 (dd, *J* = 8.0, 1.9 Hz, 2H), 6.62–6.57 (m, 2H), 5.49 (br, 1H), 5.35 (br, 2H), 3.83 (s, 3H), 2.78 (t, *J* = 3.3 Hz, 4H). ^13^C NMR (101 MHz, CDCl_3_) δ 146.3, 143.6, 143.4, 141.6, 135.0, 133.8, 121.0, 121.0, 115.7, 115.2, 114.2, 111.2, 55.9, 37.7, 37.5. LC/MS (ESI): 259.0 [M-H]^-^. HPLC purity: 96.3%.

#### 4-(3,5-dimethoxyphenethyl)-2-methoxyphenol (14d)

Compound **14d** was synthesized according to the general procedure 2. The crude product was purified by column chromatography (petroleum ether: ethyl acetate = 10:1) to yield **14d** (0.10 g, 90%) as a blue oil. ^1^H NMR (400 MHz, CDCl_3_) δ 6.83 (d, *J* = 8.0 Hz, 1H), 6.69 (dd, *J* = 8.0, 1.7 Hz, 1H), 6.63 (d, *J* = 1.6 Hz, 1H), 6.32 (dd, *J* = 7.4, 2.0 Hz, 3H), 5.54 (br, 1H), 3.83 (s, 3H), 3.76 (s, 6H), 2.83 (s, 4H). ^13^C NMR (101 MHz, CDCl_3_) δ 160.7, 146.3, 144.2, 143.8, 133.7, 121.0, 114.3, 111.2, 106.6, 98.0, 55.9, 55.3, 38.6, 37.4. LC/MS (ESI): 289.1 [M+H]^+^. HPLC purity: 96.9%.

#### 4-chloro-2-(4-hydroxy-3-methoxyphenethyl)phenol (14e)

Compound **14e** was synthesized according to the general procedure 2. The crude product was purified by column chromatography (petroleum ether: ethyl acetate = 4:1) to yield **14e** (0.05 g, 46%) as a colorless oil. ^1^H NMR (400 MHz, CDCl_3_) δ 7.06 (d, *J* = 1.6 Hz, 1H), 7.03 (d, *J* = 8.5 Hz, 1H), 6.84 (d, *J* = 8.0 Hz, 1H), 6.68 (t, *J* = 8.3 Hz, 2H), 6.61 (s, 1H), 5.52 (br, 1H), 4.82 (br, 1H), 3.82 (s, 3H), 2.83 (s, 4H). ^13^C NMR (101 MHz, CDCl_3_) δ 152.3, 146.4, 143.9, 133.4, 130.1, 129.8, 127.0, 125.4, 121.0, 116.7, 114.4, 111.2, 55.9, 35.7, 32.5. LC/MS (ESI): 277.0 [M-H]^-^. HRMS calcd for C_15_H_15_O_3_Cl [M-H]^-^: 277.0637, found: 277.0644. HPLC purity: 99.9%.

#### 4-[2-(3-hydroxy-5-methoxyphenyl)ethyl]-2-methoxyphenol (14f, gigantol)

Compound **14f** was synthesized according to the general procedure 2. The crude product was purified by column chromatography (petroleum ether: ethyl acetate = 5:1) to yield gigantol (0.50 g, 68%) as a white solid. ^1^H NMR (400 MHz, CDCl_3_) δ 6.83 (d, *J* = 8.0 Hz, 1H), 6.68 (dd, *J* = 8.0, 1.8 Hz, 1H), 6.62 (d, *J* = 1.8 Hz, 1H), 6.31 (s, 1H), 6.25 (d, *J* = 2.0 Hz, 2H), 5.47 (s, 1H), 3.84 (s, 3H), 3.75 (s, 3H), 2.80 (dt, *J* = 11.8, 5.9 Hz, 4H). ^13^C NMR (101 MHz, CDCl_3_) δ 160.9, 156.6, 146.3, 144.6, 143.8, 133.6, 121.0, 114.2, 111.2, 108.1, 106.9, 99.1, 55.9, 55.3, 38.3, 37.3. HPLC purity: 98.5%.

#### 3-methoxy-5-((4-methoxyphenylamino)methyl)phenol (17a)

Compound **17a** was synthesized according to the general procedure 4. The crude product was purified by column chromatography (petroleum ether: ethyl acetate = 3:1) to yield **17a** (0.19 g, 74%) as a yellow oil. ^1^H NMR (400 MHz, CDCl_3_) δ 6.77 (d, *J* = 8.9 Hz, 2H), 6.59 (d, *J* = 8.9 Hz, 2H), 6.49 (s, 1H), 6.42 (s, 1H), 6.29 (s, 1H), 4.18 (s, 2H), 3.75 (s, 3H), 3.73 (s, 3H). ^13^C NMR (101 MHz, CDCl_3_) δ 161.1, 157.3, 152.4, 142.3, 142.2, 115.0, 114.8, 107.0, 105.5, 100.3, 55.9, 55.3, 49.4. LC/MS (ESI): 260.1 [M+H]^+^. HRMS calcd for C_15_H_17_NO_3_ [M+H]^+^: 260.1281, found: 260.1287. HPLC purity: 95.3%.

#### 4-((3,4-dimethoxyphenethylamino)methyl)phenol (17b)

Compound **17b** was synthesized according to the general procedure 4. The crude product was purified by column chromatography (petroleum ether: ethyl acetate = 5:1) to yield **17b** (0.24 g, 82%) as a white solid. mp: 116.7–118.6°C. ^1^H NMR (400 MHz, CDCl3) δ 7.05 (d, *J* = 8.5 Hz, 2H), 6.81 (d, *J* = 8.1 Hz, 1H), 6.76–6.72 (m, 2H), 6.62 (d, *J* = 8.5 Hz, 2H), 3.87 (s, 3H), 3.84 (s, 3H), 3.71 (s, 2H), 2.93 (t, *J* = 7.0 Hz, 2H), 2.81 (t, *J* = 6.9 Hz, 2H). ^13^C NMR (101 MHz, CDCl_3_) δ 156.3, 149.0, 147.5, 131.9, 129.7, 129.6, 120.6, 115.9, 112.0, 111.5, 55.9, 55.8, 53.2, 50.2, 35.1. LC/MS (ESI): 288.1 [M+H]^+^. HPLC purity: 98.0%.

#### 2-((benzo[d]thiazol-2-ylamino)methyl)-5-methoxyphenol (17c)

Compound **17c** was synthesized according to the general procedure 4. The crude product was purified by column chromatography (petroleum ether: ethyl acetate = 4:1) to yield **17c** (0.19 g, 65%) as a white solid. mp: 184.3–185.7°C. ^1^H NMR (400 MHz, MeOD) δ 7.57 (dd, *J* = 7.9, 0.7 Hz, 1H), 7.44 (d, *J* = 7.6 Hz, 1H), 7.26 (td, *J* = 7.8, 1.2 Hz, 1H), 7.16 (d, *J* = 8.8 Hz, 1H), 7.06 (td, *J* = 7.8, 1.1 Hz, 1H), 6.48–6.36 (m, 2H), 4.49 (s, 2H), 3.74 (s, 3H). ^13^C NMR (101 MHz, CDCl_3_) δ 167.8, 160.8, 156.8, 150.4, 131.5, 129.7, 126.0, 121.9, 120.8, 118.1, 117.8, 106.2, 103.2, 55.2, 44.1. LC/MS (ESI): 287.0 [M+H]^+^. HRMS calcd for C_15_H_14_N_2_O_2_S [M+H]^+^: 287.0849, found: 287.0863. HPLC purity: 97.6%.

#### (*E*)-3-(3,5-dimethoxyphenyl)-1-(4-fluorophenyl)prop-2-en-1-one (19)

A mixture of 1-(4-fluorophenyl)ethanone (2 mmol) and 3,5-dimethoxybenzaldehyde (2.2 mmol) in ethanol was added to K_2_CO_3_ (20 mmol) and stirred at room temperature overnight. After thorough extraction with EtOAc, the combined organic layer was washed with brine, dried over anhydrous sodium sulfate, filtered, and concentrated under reduced pressure. The resulting crude compound **19** was purified using silica gel column chromatography with EtOAC/petroleum ether (1/20) as eluent to produce 0.51 g yellow oil, yield: 89%. ^1^H NMR (400 MHz, CDCl_3_) δ 8.05 (t, *J* = 6.0 Hz, 2H), 7.72 (d, *J* = 15.7 Hz, 1H), 7.44 (d, *J* = 15.7 Hz, 1H), 7.18 (t, *J* = 7.9 Hz, 2H), 6.77 (s, 2H), 6.54 (s, 1H), 3.84 (s, 6H).

#### 1-(3-(4-fluorophenyl)propyl)-3,5-dimethoxybenzene (20)

Compound **20** was synthesized according to the general procedure 2. The crude product was purified by column chromatography (petroleum ether: ethyl acetate = 10:1) to yield **20** (0.23 g, 48%) as a colorless oil. ^1^H NMR (400 MHz, CDCl_3_) δ 7.18–7.01 (m, 2H), 7.00–6.85 (m, 2H), 6.43–6.17 (m, 3H), 3.74 (s, 6H), 2.69–2.42 (m, 2H), 2.71–2.38 (m, 2H), 2.13–1.65 (m, 1H), 2.05–1.79 (m, 1H).

#### 5-(3-(4-fluorophenyl)propyl)benzene-1,3-diol (21)

Compound **21** was synthesized according to the general procedure 3. The crude product was purified by column chromatography (petroleum ether: ethyl acetate = 4:1) to yield **21** (0.19 g, 92%) as a yellow oil. ^1^H NMR (400 MHz, CDCl_3_) δ 7.06 (s, 2H), 6.92 (t, *J* = 8.4 Hz, 2H), 6.22 (s, 2H), 6.18 (s, 1H), 2.55–2.51 (m, 2H), 2.45–2.41 (s, 2H), 1.81 (d, *J* = 4.7 Hz, 2H). ^13^C NMR (101 MHz, CDCl_3_) δ 162.4, 160.0, 156.4, 145.7, 137.7, 137.7, 129.7, 127.7, 115.1, 114.9, 108.3, 100.4, 35.1, 34.4, 32.4. LC/MS (ESI): 247.1 [M+H]^+^. HRMS calcd for C_15_H_15_O_2_F [M-H]^-^: 245.0983, found: 245.0987. HPLC purity: 98.8%.

#### *N*-(4-hydroxyphenyl)-4-methylbenzenesulfonamide (23)

Compound **23** was synthesized according to the general procedure 3. The crude product was purified by column chromatography (petroleum ether: ethyl acetate = 5:1) to yield **23** (0.17 g, 63%) as a yellow oil. ^1^H NMR (400 MHz, MeOD) δ 7.31 (d, *J* = 8.2 Hz, 2H), 7.04 (d, *J* = 7.9 Hz, 2H), 6.61 (d, *J* = 8.8 Hz, 2H), 6.38 (d, *J* = 8.8 Hz, 2H), 3.15–3.03 (m, 3H). ^13^C NMR (101 MHz, CDCl_3_) δ 159.2, 147.3, 140.4, 133.0, 132.7, 130.9, 128.9, 119.1, 24.0. LC/MS (ESI): 262.0 [M-H]^-^. HPLC purity: 99.8%.

#### *N*-(3,4-dimethoxyphenethyl)-2-(4-hydroxyphenyl)acetamide (25)

2-(4-hydroxyphenyl)acetic acid (1.0 mmol) and 2-(3,4-dimethoxyphenyl) ethanamine (1.0 mmol) were mixed and stirred at 180°C for 4 hours under N_2_. Crude product **25** was purified by recrystallization using acetonitrile to produce 0.25 g gray solid. Yield: 80%, mp: 151.7–153.2°C. ^1^H NMR (400 MHz, MeOD) δ 7.02 (d, *J* = 8.4 Hz, 2H), 6.80 (dd, *J* = 15.8, 4.9 Hz, 2H), 6.71 (d, *J* = 8.5 Hz, 2H), 6.68–6.62 (m, 1H), 3.80 (s, 3H), 3.78 (s, 3H), 3.39 (t, *J* = 7.1 Hz, 2H), 3.35 (s, 2H), 2.71 (t, *J* = 7.0 Hz, 2H). ^13^C NMR (101 MHz, MeOD) δ 174.6, 157.5, 150.4, 149.1, 133.3, 131.1, 127.6, 122.3, 116.4, 113.8, 113.2, 56.6, 55.4, 43.2, 42.0, 35.9. LC/MS (ESI): 316.1 [M+H]^+^. HPLC purity: 98.1%.

### Biological assay

#### AR inhibition assays

AR was purchased from Prospec-TanyTechnogene Ltd. (Israel). AR activity was evaluated as previously described [48, 49]. Briefly, the reaction mixture was composed of enzyme solution (20 mmol·L^-1^, 20 μL), NADPH (0.104 mmol·L^-1^, 50 μL; Italian Roth, Italy), DL-glyceraldehyde (10 mmol·L^-1^, 50 μL; Sigma, U.S.), buffer phosphate (0.1 mol·L^-1^), and selected concentrations of test compounds. The reaction was initiated by the addition of DL-glyceraldehyde as the substrate, and PBS was used as the blank control. The use of NAPDH, in parallel to AR activity, was monitored at room temperature for 10 min at 40 s intervals at 340 nm in an EnSpire^™^ Multimode Plate Reader. AR inhibition was expressed as inhibition rate (%) = [1−(A_2_ − A_0_)/(A_1_ − A_0_)] ×100% [50], where A_0_ represents the decrease in NADPH absorbance without AR, substrate, or compounds; A_1_ represents the decrease in NADPH absorbance prior to the test compounds addition; A_2_ represents decrease in NADPH absorbance following the test compounds addition. All assays were performed in triplicate.

#### iNOS inhibition assays

iNOS produces NO by catalyzing a reaction involving L-Arg and oxygen. Its activity was assessed by monitoring NO production using a Nitric Oxide Synthase Assay Kit (Nanjing Jiancheng Bioengineering Institute, China), which was developed based on the method of Fröhlich et al. [51]. The magnitude of iNOS inhibition is expressed as inhibition rate which was calculated using the following equation: (%) = [(B_1_ − B_2_)/(B_1_ − B_0_)] × 100%, B_0_ and B_1_ represented the absorbance values obtained for the blank (control solution) and standard, respectively, and B_2_ represented the absorbance of the test compounds. All assays were performed in triplicate.

#### Cell viability assays

Human lens epithelial cells (HLECs; SRA 01/04) were obtained from Dr. Fu Shang, USDA HNRCA at Tufts University, Boston, MA, U.S. and Zhongshan Ophthalmic Center, Sun Yat-sen University, Guangzhou, P. R. China [52, 53] and cultured in minimal essential medium (MEM) supplemented with 20% fetal bovine serum (FBS) and a cocktail of Penicillin-Streptomycin at 37°C in a humid environment containing 5% CO_2_ [52]. Cells were harvested at 80% confluency by trypsinization, and then fresh culture medium was added to generate single-cell suspensions for use in cell viability assays. HLECs were then seeded in 96-well cell culture grade microplates at a density of 1 × 10^5^·mL^-1^. After 24 h of incubation, the cells were treated for 72 h with 250 mmol·L^-1^ D-galactose with and without test compounds (0.1, 0.5, or 1.0 μg·mL^-1^) [45].

Cell viability was assessed using an MTT assay. After the treatments, 20 μL of 5 mg·mL^-1^ MTT solution was added to each well, followed by incubation for 4 h at 37°C. The resulting formazan crystals were then dissolved in 150 μL DMSO and absorbance measured at 570 nm using an EnSpire^™^ Multimode Plate Reader. The results are presented as percentages of cell survival as compared to the untreated control group, and all assays were performed in triplicate.

#### Statistical methods

Statistical analyses and data processing were performed using the SPSS v.16.0 statistical software. Cell viability data are presented as mean ± SD. One-way ANOVA was performed to assess statistical significance between the test compounds. *P* < 0.05 was considered statistically significant.

## Supporting Information

S1 File Contents: ^1^H and ^13^C NMR spectra of target compounds. (DOCX)
