# Supplementary material for: Synthesis and Biological Evaluation of Novel Gigantol Derivatives as Potential Agents in Prevention of Diabetic Cataract
Source: PLoS One. 2015 Oct 30;10(10):e0141092. doi: 10.1371/journal.pone.0141092 (PMC4627826; doi:10.1371/journal.pone.0141092)
Supplement: S1 File — (DOCX) [file pone.0141092.s001.docx]

**Supporting Information**

**Synthesis and biological evaluation of novel gigantol derivatives as potential agents in prevention of diabetic cataract**

Jie Wu^1‡^, Chuanjun Lu^2,3‡^, Xue Li^1^, Hua Fang^1^, Wencheng Wan^1^, Qiaohong Yang^1^, Xiaosheng Sun^1^, Meiling Wang^1^, Xiaohong Hu^1^, C-Y. Oliver Chen^4^, Xiaoyong Wei^1,4*^

1 School of Basic Medical Sciences, Guangzhou University of Chinese Medicine, Guangzhou 510006, China

2 College of Chemical Engineering, Zhejiang University of Technology, Hangzhou 310014, China

3 Institute of Drug Synthesis and Pharmaceutical Processing, School of Pharmaceutical Sciences, Sun Yat-sen University, Guangzhou 510006, China

4 Antioxidants Research Laboratory, Jean Mayer USDA Human Nutrition Research Center on Aging, Tufts University, Boston, MA 02111, U.S.A

**^‡^**Jie Wu and Chuanjun Lu contributed equally to this work

*Corresponding author:

Xiaoyong Wei

Tel: +1-617 309 0520

E-mail address: jidewowxy@163.com

**^1^H and ^13^C NMR spectra of target compounds.**

^1^H NMR spectra of compound **4**

^13^C NMR spectra of compound **4**

^1^H NMR spectra of compound **5**

^13^C NMR spectra of compound **5**

^1^H NMR spectra of compound **8**

^13^C NMR spectra of compound **8**

^1^H NMR spectra of compound **10**

^13^C NMR spectra of compound **10**

^1^H NMR spectra of compound **14a**

^13^C NMR spectra of compound **14a**

^1^H NMR spectra of compound **14b**

^13^C NMR spectra of compound **14b**

^1^H NMR spectra of compound **14c**

^13^C NMR spectra of compound **14c**

^1^H NMR spectra of compound **14d**

^13^C NMR spectra of compound **14d**

^1^H NMR spectra of compound **14e**

^13^C NMR spectra of compound **14e**

^1^H NMR spectra of compound **14f**

^13^C NMR spectra of compound **14f**

^1^H NMR spectra of compound **17a**

^13^C NMR spectra of compound **17a**

^1^H NMR spectra of compound **17b**

^13^C NMR spectra of compound **17b**

^1^H NMR spectra of compound **17c**

^13^C NMR spectra of compound **17c**

^1^H NMR spectra of compound **21**

^13^C NMR spectra of compound **21**

^1^H NMR spectra of compound **23**

^13^C NMR spectra of compound **23**

^1^H NMR spectra of compound **25**

^13^C NMR spectra of compound **25**
